# Phytochemical Profiling and Toxicity Assessment of Aqueous Extract From Bitter Apricot Kernels Cultivated in Morocco

**DOI:** 10.1155/sci5/5757744

**Published:** 2025-02-18

**Authors:** Mohamed Amine El-Hajjaji, Ghizlane Nouioura, Kawtar Fikri-Benbrahim, Najoua Soulo, Mohammed El Ouassete, Badiaa Lyoussi, Zineb Benziane Ouaritini

**Affiliations:** ^1^Department of Biology, Faculty of Sciences, Sidi Mohamed Ben Abdellah University, Fez, Morocco; ^2^Department of Biology, Faculty of Sciences and Technologies, Sidi Mohamed Ben Abdellah University, Fez, Morocco

**Keywords:** acute toxicity, phytochemicals, *Prunus armeniaca*. L, sub-acute toxicity

## Abstract

Apricot kernels contain amygdalin, a cyanogenic glycoside that degrades to cyanide upon chewing or crushing, posing a potential toxicity risk to humans. The present study aimed to determine the phenolic compounds and to evaluate the subacute and acute toxicity of the aqueous extract of bitter apricot kernels (BAKs) in Swiss albino mice. The chemical characterization was carried out with HPLC-DAD analyses, and acute toxicity was done by extract's oral administration once for 72-h period at doses of 500–6000 mg/kg body weight (bw). For the subacute toxicity, mice were administrated orally by repeated doses of 100, 500, and 1000 mg/kg bw for 28 days. The hematological, biochemical parameters and the histological examinations of vital organs (kidney, liver, and spleen) were done by sacrificing the animals after the subacute toxicity period. The results revealed 11 phenolic compounds with a total of 61 mg/g of extract. In the acute toxicity study, no signs of toxicity or mortality were observed during the experiment period, and the LD_50_ value was higher than 6000 mg/kg bw. In the subacute toxicity, only the group treated with the greatest dose (1000 mg/kg bw) exhibited a significant decrease in the hematocrit and slight increase in urea, and creatinine. The results of this study indicate that the aqueous extract of BAK was not toxic to mice at the tested concentrations. This provides valuable information regarding its toxicity profile.

## 1. Introduction

Natural resources have long been used as remedies for preventing or treating various diseases due to their potent therapeutic effects and affordability [1]. People often choose natural products, believing them to be safe and free from adverse effects [2]. Despite the complex secondary metabolites present in medicinal plants, herbs are frequently used to treat specific ailments without sufficient scientific evidence or understanding of their potential toxicological effects [3]. Chemical safety is determined by analyzing the potential damage and mechanisms of action of substances through the use of acute and subacute toxicity studies. Acute toxicity assays, which are based on single-dose administration, help to identify symptoms and analyze the effects of toxicity on animals. Subsequent subacute toxicity studies, involving repeated doses, are conducted after initial insights from acute toxicity tests, providing valuable information about the specific target tissues or organs affected in animals [4].

Apricot belong to the Rosaceae family, which is native to China and Japan and cultivated in various regions worldwide, such as Morocco, Pakistan, Turkey, Spain, Iran, the United States, France, Italy, Australia, and Russia [5]. Apricot kernels are rich in phytochemicals such as polyphenolics, carotenoids, and glycosides, along with essential nutrients such as carbohydrates; Vitamins A, C, and E; minerals; and fibers. Apricots are known for their diverse phenolic content, including compounds such as catechin, epicatechin, *p*-coumaric acid, caffeic acid, ferulic acid, and their esters [6, 7]. Research has indicated that apricot kernels possess numerous pharmacological benefits, demonstrating properties such as anti-inflammatory, antimicrobial, antiparasitic, anticancer, antioxidant, hepatoprotective, and cardioprotective [5].

Apricot kernels contain cyanogenic glycosides (amygdalin), which are classified as antinutritional due to their conversion into the toxic compound hydrocyanic acid. Its content in the kernels typically ranges from 3%–4% to 8% [8]. Certain varieties, referred to as bitter apricots, exhibit elevated levels of these cyanogenic compounds, with concentrations ranging from around 240–350 mg of hydrocyanic acid per 100 g. Conversely, sweet apricots contain minimal amounts of these compounds [9].

The decoction is the most used preparation method by the Moroccan population with a percentage of 33% [3]. Ground apricot kernels are often used to enhance the flavor of pastries and cakes in many regions. Similarly, bitter almonds are widely utilized in making traditional orgeat syrup (almond syrup), a popular and commonly enjoyed beverage ingredient [10].

To the best of our knowledge, no published studies have examined the bioactive compounds and the toxicity of *Prunus armeniaca* L. kernels grown in Morocco. This study aimed to determine the phytochemical profile and evaluate the subacute and acute oral toxicity in mice of the aqueous extract from Moroccan apricot kernels to better predict potential risks and ensure safe usage in humans.

## 2. Material and Methods

### 2.1. Plant Material

The apricot kernels of the Maoui variety were sourced from the Sefrou region in Morocco (33°40′13.6″N 4°51′28.7″W) in May 2023. The sample was identified by botanists from Sidi Mohamed Ben Abdellah University (USMBA) and registered under voucher code RPA 001 VM 2326 SL.

### 2.2. Extraction Method

The traditional method of extracting chemical ingredients in medicinal materials involves decoction in boiling water [11]. Briefly, 100 g of ground BAKs was boiled in 1 L of distilled water at 100°C for 20 min, slightly lowering the temperature after boiling to prevent excessive degradation of the active compounds. After cooling at room temperature for approximately 30 min, the mixture was filtered through Whatman No. 1 paper. The resulting filtrate was then dried in a drying oven/incubator (BOV-D35, BIOBASE) set at 35°C for two days. The resulting extract (yield: 6.25% w/w) was stored at 4°C for future experiments.

## 3. Animals

Male and female Swiss mice (30 ± 2 g) were provided by the animal breeding center of the Faculty of Sciences, USMBA, Fez, Morocco. The mice were acclimatized during the experimental period in a controlled environment, kept out at 12 h light/dark cycle, adequate ventilation, 25°C ± 2°C of temperature, and unrestricted nourishment access. Animals were used according to the National Research Council's Guide for the Care and Use of Laboratory Animals' guidelines. The experimental procedure followed in the present study received approval from the Ethical Committee of the Faculty of Sciences and Techniques of Sidi Mohamed Ben Abdellah University (Ethical Approval Number: 01/2024/CEFST). At the end of the experiments, animal's euthanasia was performed by decapitation, a common method for the humane termination of small laboratory animals. Prior to decapitation, the animals were anesthetized with sodium pentobarbital, administered by intraperitoneal route at a dose of 30 mg/kg bw. Decapitation was conducted using a guillotine specifically designed for laboratory rodents. The guillotine was calibrated and maintained to ensure a swift and clean cut, providing immediate cessation of brain activity.

### 3.1. Phenolic Compounds of Bitter Apricot Kernel (BAK) Extracts

Phenolic compounds present in the studied extract were identified and quantified in accordance with the method of the International Olive Oil Council [12], with some changes. High-performance liquid chromatography equipped with a diode array detector (HPLC-DAD), an LC A20 quaternary pump, a degasser, and a temperature-controlled autosampler were employed. Polyphenols were separated by HPLC on a Supelco C18 Ascentis C18 analytical column (4.6 × 150 mm, 5 µm, 100A), with a flow rate of 0.5 mL/min. The mobile phase consisted of a ternary mixture of acetonitrile, methanol, and acidified water (0.2% glacial acetic acid buffer). A 45°C column temperature was maintained, and a 10 μL injection volume was used. Standard solutions of gallic acid and tyrosol were injected under the same conditions. The results were reported in milligrams per gram dry weight of extract (mg/g of extract).

### 3.2. Acute Toxicity

A total of 40 mice (Swiss albino) of both sexes (male and female), randomly divided into eight groups of five each (*n* = 5), with an individual body weight of 30 ± 2 g, underwent oral administration of doses of 500, 1000, 2000, 3000, 4000, 5000, and 6000 mg/kg bw, while the control group received only normal saline solution (0.09% NaCl). Subsequent to the oral administration of the extracts, the manifestation of toxic symptoms and the mortality rate within each group were meticulously observed and recorded over a 72-h period. The determination of LD_50_ values was executed following the methodology outlined by Litchfield et Wilcoxon [13], and Guidance OECD 423 in the acute toxicity test [14]. The acute toxicity test has been conducted according to the standard practices for initial toxicity evaluation [14].

### 3.3. Subacute Toxicity

A total of 20 Swiss albino mice, comprising both males and females, were divided into four groups. During the 28-day experiment, mice of the control group were given only normal saline solution (0.9% NaCl); while the other groups were administered orally the studied extract at doses of 100, 500, and 1000 mg/kg bw, respectively [15]. It is to note that starting with a lower dose in the subacute study reduces the risk of severe toxicity and allows for a more detailed evaluation of the compound's long-term effects at moderate levels as the effects in this case were assessed over a prolonged period (28 days) with repeated administrations.

### 3.4. Hematological Parameters

The hematological analysis of hematocrit (HCT), hemoglobin (HGB), red blood cell (RBC), and white blood cell (WBC) concentration was done by collecting 300 mL of blood into a “complete blood content” (CBC) bottle containing the anticoagulant tripotassium ethylenediamine tetra-acetic acid. These parameters were determined using an automated blood analyzer (the Sysmex KX-21 (Sysmex Corp., Japan)).

### 3.5. Biochemical Parameters

Various biochemical parameters, such as aspartate aminotransferase (AST), alanine aminotransferase (ALT), blood urea nitrogen (BUN), and creatinine (CRE) were measured by isolating the serum from a 0.5 mL blood sample after centrifugation at 3000 rpm for 10 min. These parameters were analyzed with biochemistry analyzer (Beckman Coulter, Tokyo, Japan).

### 3.6. Histopathology Evaluation

After the subacute toxicity study period, the animals underwent euthanasia, and a gross necropsy was conducted. The kidneys, spleen, and liver underwent macroscopic examination. Preservation of the organs was accomplished within 48 h using a 10% buffered formalin solution (pH 7.4). The organs were fixed in paraffin, and tissue sections with a thickness of 5 μm were produced using a rotating microtome. For later microscopic inspection, these sections were stained with hematoxylin and eosin.

### 3.7. Statistical Analysis

Data analysis was performed by using GraphPad Prism version 8.0 for Windows. All results are presented as means ± standard deviation (SD). The statistical multiple comparison between groups was done through a one-way ANOVA and then Dunnett's test.

## 4. Results

### 4.1. Phenolic Compounds of BAK Extracts

**Figure 1:**
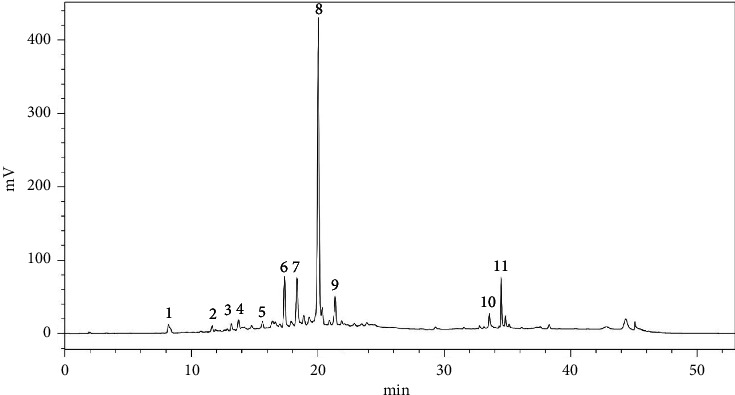
Chromatogram of phenolic compounds of bitter apricot kernel extracts.

The HPLC analysis results indicated the identification of 11 distinct phenolic compounds within the sample as shown in Figure 1 with a total of 60.31 ± 0.11 mg/g of extract. Among these, procyanidin emerged as the predominant compound, suggesting its significant presence and potential influence on the overall phenolic profile. Following procyanidin, epicatechin was identified as the next most abundant compound, further contributing to the sample's phenolic content. In contrast, compounds such as salicylic acid, vanillic acid, and gallic acid were detected in minor quantities (Table 1, Figure 1).

### 4.2. Acute Toxicity

Within the scope of the acute toxicity assay, our investigation yielded significant findings over the course of the 3 day's evaluation period. Mice orally administered of the aqueous extract showed no toxic symptoms such as convulsions, ataxia, diarrhea, or increased diuresis, and no deaths were recorded among the tested animals exposed to various doses from 500 to 6000 mg/kg bw of the extract. The median lethal dose (LD_50_) for the tested sample was found to be higher than the highest dose given to the treated mice, which was 6000 mg/kg bw.

### 4.3. Subacute Toxicity

#### 4.3.1. Body Weights of Mice

**Figure 2:**
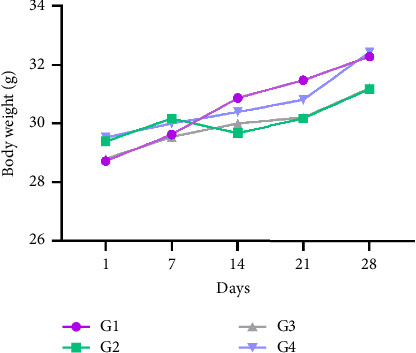
Variation in mice body weight during a 28-day period. G1: control group; G2: group treated with 100 mg/kg bw; G3: group treated with 500 mg/kg bw; G4: group treated with 1000 mg/kg bw.

During the subacute toxicity test period, the mice body weights were measured once a week. The results revealed no significant differences (*p* > 0.05) in the body weight changes between the control group (G1) and other groups administrated orally the doses of 100–1000 mg/kg bw, respectively (Figure 2).

### 4.4. Organ Weights of Mice

**Figure 3:**
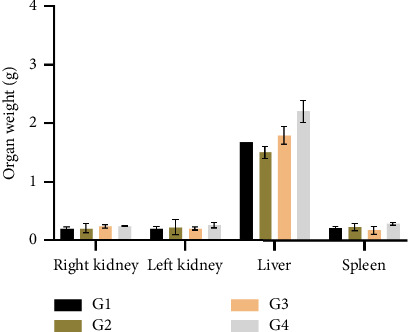
Change in the organ weight of mice. G1: control group; G2: group treated with 100 mg/kg bw; G3: group treated with 500 mg/kg bw; G4: group treated with 1000 mg/kg bw.

The weights of the primary organs, including kidneys, liver, and spleen, were assessed after 28 days of therapy with the investigated extract in mice. The organ weights did not significantly change (*p* > 0.05) between the control and the treated groups (Figure 3).

### 4.5. Hematological Parameters

The hematological parameters, RBC, HGB, HCT, and WBC, impacted by the investigated extract during the subacute test are compiled in Table 2. For the second and third groups that received oral dosages of 100 and 500 mg/kg bw, respectively, no statistically significant change (*p* > 0.05) was recorded in RBC, HGB, WBC, and HCT when compared to the control group. Moreover, the HCT significantly decreased (*p* < 0.05) in the fourth group, which received 1000 mg/kg bw.

### 4.6. Biochemical Parameters

The obtained results show that the liver marker enzymes (ALT and AST) did not change significantly by the daily ingestion of the studied extract for 28 days at any dose. In contrast, the group (G4) that received 1000 mg/kg bw treatment showed a notable rise in urea and creatinine (CRE) levels (*p* < 0.05) (Table 3).

### 4.7. Histological Analysis

**Figure 4:**
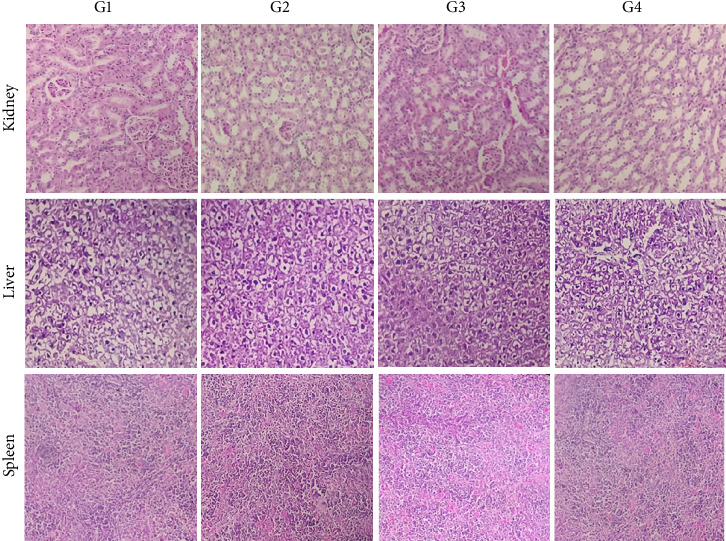
Histological analyses of the kidney, liver, and spleen tissues of mice. G1: control group; G2: group treated with 100 mg/kg bw; G3: group treated with 500 mg/kg bw; G4: group treated with 1000 mg/kg bw.

The results presented in Figure 4 show that the kidney, liver, and spleen tissues of mice given an oral dose of the studied extract (100–1000 mg/kg bw) exhibited normal morphology and no appreciable changes in the cellular architecture compared to the control group have been seen.

## 5. Discussion

Consuming cyanogenic substances from some plants, such as almonds, cherries, plums, peaches, and apricots, can result in both acute and subacute health issues, including headaches, nausea, vomiting, and cardiac arrest [16]. BAKs possess several bioactive compounds that have multiple biological activities and are used to treat various diseases [17].

The phytochemical profile of the studied extract includes various flavonoids, phenolic acids, anthocyanins, and other phytochemicals. Procyanidin was identified as the predominant compound, highlighting its significant presence in the extract, and epicatechin was the second most abundant compound. The identification of these compounds, particularly, the high levels of procyanidin and epicatechin, underscores the potential health benefits of BAK extracts. Flavonoids, known for their antioxidant, anti-inflammatory, and cardioprotective properties, play a crucial role in promoting overall health [18]. Phenolic acids contribute additional antioxidant activity, while anthocyanins are recognized for their anti-inflammatory and anticancer properties [19, 20].

Procyanidin, a polyphenol found abundantly in nature, is made up of flavan-3-ol units like catechin and epicatechin, it is commonly found in cranberries, apples, pine bark, tea, cocoa, and grapes. Procyanidin exhibits a variety of beneficial properties, including antioxidant, anticancer, antitumor, immunosuppressive, and antiallergy effects and in the protection against metabolic disorders and chronic diseases [21, 22].

One of the most common secondary metabolites in plants and a major source of plant polyphenols in the diet is epicatechin [23]. It has several health benefits, such as antioxidant and anti-inflammatory properties, improved muscular performance, reduced symptoms of cerebrovascular and cardiovascular disorders, diabetes prevention, and nervous system protection [24].

Cyanidin 3-O-galactoside is one of the most prevalent anthocyanins, known for its positive impact on the health of both animals and humans. Its purified form, as well as its combination with other polyphenols, exhibits notable antioxidant properties and several health benefits, especially antidiabetic, anticancer, antitoxic, anti-inflammatory, cardiovascular, and neuroprotective effects [25].

D-mandelonitrile-beta-D-gentiobioside (amygdalin), the primary cyanogenic glycoside in Prunus species, was used in ancient times to treat various infections such as leukoderma, nausea, asthma, and leprosy [26]. Amygdalin's pharmacological properties have been thoroughly established throughout the years and include anti-inflammatory, antifibrotic, analgesic, immunoregulatory, auxiliary anticancer, antiatherosclerotic, anticardiac hypertrophy, antiulcer, and hypoglycemic effects [27]. The current study indicated the presence of 4.1 ± 0.2 mg/g of extract of amygdalin in the studied extract. In a study conducted by Bolarinwa, Orfila, and Morgan [16], apricot kernels were found to contain a high concentration of amygdalin (14.37 ± 0.28 mg/g). Furthermore, the amygdalin contents of non-Rosaceae seeds from marrow, melon, zucchini, and cucumber were, respectively, 0.06, 0.07, 0.12, and 0.21 mg/g. It was outlined that the toxicity in humans occurs when amygdalin is administered orally at 4 g per day for 15 days or through intravenous injection for a month. However, reducing the dose to daily oral doses can help avoid toxicity [28].

The acute toxicity test revealed the absence of death and any toxic symptoms, and the median lethal dose (LD_50_) for the tested sample was found to be higher than 6000 mg/kg bw. In terms of toxicity classification, chemicals that have LD_50_ values of more than 5000 mg/kg bw are often regarded as nontoxic [29]. The findings showed that the oral administration of the BAK' aqueous extract prepared by the decoction method is nontoxic at the selected doses. Our findings align with those of Ramadan et al. [6], who similarly reported the absence of deaths and the absence of toxic symptoms in animals orally administrated the tested doses of 70%, and 99% ethanolic extracts of apricot seeds during the experimental period (72 h), and the LD_50_ was to be higher than 10 g/kg bw for both extracts. Furthermore, prior research conducted by Badr, Tawfik, and Badr [30] revealed that the oral administration of apricot kernel water extract at doses ranging from 5 to 100 mg/kg bw, resulted in no significant toxic signs or death in all subjected animals during the 72-h observation period, and the median lethal dose (LD_50_) was found to exceed the highest dose orally administrated. This LD_50_ assessment not only underscores the absence of acute lethality associated with the tested substances but also reaffirms the relative safety margin observed across all tested dose levels. In addition, Sisodia, Sharma, and Singh [31] investigated the potential toxicity of *P. avium* (Cherry), a member of the Rosaceae family. The study discovered that after 15 days of oral administration of the methanolic extract of cherry fruit, the LD_50/15_ of this extract was 4.947 mg/kg bw.

After enzymatic hydrolysis, amygdalin generates significant amounts of hydrocyanic acid and benzaldehyde, which contribute to the bitter taste of Prunus species [26]. Although apricot kernels contain elevated levels of HCN, boiling the seeds has shown to reduce 90%–100% of hydrocyanic acid [32]. Additionally, research indicates that the duration and temperature of the extraction process significantly contribute to removing bitterness from the apricot kernel [33]. In addition, amygdalin was extracted from almond kernels in boiling water and showed an increase in the yield over time and the maximum yield after 100 min (6.8 mg/100 g), after which a decline in yield was observed [16]. These discoveries can help to explain the fact that our extract, which was prepared by decoction method, displayed no indications of toxicity or mortality upon oral administration. These robust results collectively point to the commendable safety profile of the investigated samples, providing valuable insights into their potential use and application in various contexts.

Even at moderate doses, the initially nontoxic substance may become toxic over time due to the accumulation and disturbance of physiological and metabolic balance. There was no statistically significant difference (*p* > 0.05) in the body weight of mice treated with the studied extract daily via the oral route for 28 days in the current investigation. This lack of significant difference suggests that the administered apricot kernel extracts did not induce any discernible adverse effects on the overall weight of the mice. Indeed, the decrease in body weight is a common sensitive indicator of toxicity [29]. Organ weights have also been shown in earlier studies to be a valuable indicator of an animal's health and well-being [34]. The liver, kidneys, and spleen organ weights of the treatment and control groups did not differ significantly, according to the current study. However, it is noteworthy that only 1 mouse, belonging to the highest dosage group (1000 mg/kg bw), succumbed to the extract during the course of the study. Despite this isolated incident, the overall stability in body and organ weights across the treated groups indicates a generally well-tolerated subacute exposure to apricot kernel extracts in mice.

Evaluating biochemical parameters related to liver and kidney function is also crucial for assessing the toxicity of substances or plant extracts. Transaminases, such as AST and ALT, are particularly important enzymes indicative of liver function and are commonly used as biomarkers for potential toxicity. ALT is generated by liver cells and levels rise during inflammation or cell death. As cells are destroyed, ALT leaks cause an increase in serum levels. It is the most sensitive measure of liver cell injury. Elevated plasma amounts of AST and ALT, released by the liver, indicate hepatic injury [35].

The current study found that these markers in the treated groups did not show significant changes compared to the control group, suggesting that the extract being studied may not have caused notable toxicity to the liver. Serum urea and CRE concentrations were assessed as kidney function indices in this study. Through its conversion into urea, ammonia (NH_3_) generated during deamination was removed from the blood. The initial findings revealed a notable rise in urea levels for the group that received the largest dose of the studied extract. A significant increase in urea rates was detected in the group receiving the highest dose of the investigated extract. This increase in urea may be attributed to elevated glomerular filtration. Although CRE is typically not reabsorbed, all of the CRE filtered in the glomerular filtrate passes through the tubular system before being excreted in urine [36]. In this study, a significant increase was observed in the level of CRE in the group that received the highest dose of the studied extract. In this case, the CRE may not have been excreted in the urine but reabsorbed. In an earlier investigation by Nazneen and Naz [37], the oral administration of an aqueous extract of apricot seeds at doses ranging from 1 to 2 g/kg bw per day for 48 days showed no significant renal toxicity or hepatotoxicity.

The hematological system is responsive to harmful substances and serves as a significant gauge for tracking physiological alterations in both humans and animals [38]. The absence of statistically significant changes in the selected hematological parameters between the control and treatment groups suggests that the investigated aqueous extract had a minimal toxic impact since it was not harmful enough to cause reactions in these parameters. In a previous study by Kim, Han, and Jeon [39], the toxicity of mumefural, a bioactive compound derived from the fruit of *P. mume*, was investigated by the oral administration of single and repeated doses. The study revealed that mumefural did not cause any mortality, changes in body weight, abnormal organ damage, or alterations in hematological parameters.

Furthermore, the study conducted by Elwan et al. [40] highlighted the prophylactic effect of apricot (*P. armeniaca)* seed extract against the toxicity induced by cyclophosphamide (CTX) on kidney and liver tissues, and blood cells in Swiss mice. The study revealed that the pretreatment of mice with that extract significantly modulated the CTX-induced leukopenia. Additionally, it partially protected the liver from injury and improved kidney functions in CTX-injected mice by inducing mild improvement in their histological structure and liver antioxidant defense system. The possible impact of short-term oral apricot seed treatment on rabbit's kidney structure was highlighted in an earlier investigation by Kolesárová et al. [41]. According to the findings, there was a significant decrease in the average size of all renal structures, including the diameter of tubules, height of epithelial tubules, renal corpuscle diameter, and glomerular diameter, following 28 days of daily administration of 300 mg/kg bw of apricot seeds. Conversely, daily oral administration of apricot kernels with a dose of 60 mg/kg of bw showed no significant impact on the aforementioned parameters. Additionally, apricot oil was previously studied by Ilyas et al. [42], for its potential protective effect against liver and renal toxicity caused by mercuric chloride in rats. The findings made it abundantly evident that apricot oil bolsters the antioxidant defense mechanism in mercuric chloride-induced toxicity and offers proof that it might be useful in treating diseases caused by free radicals.

There were no signs of toxicity found in the histological assessment. The evidence for the safety of the BAK aqueous extract at the tested doses is strengthened by the agreement between the histology results and those from the hematological and biochemical investigations.

The absence of significant toxic effects suggests that the BAK, despite its content of cyanogenic glycosides, may be safe for consumption in controlled amounts. This finding is particularly relevant for regions where these kernels are traditionally used in culinary practices and herbal remedies. While the study provides valuable insights into the safety and phytochemical richness of BAK, it also opens avenues for future research.

While the current study provides valuable insights into the short-term (28-day) subacute toxicity of the extract, it is crucial to conduct long-term studies to evaluate chronic toxicity and potential delayed adverse effects. Such studies would help determine if prolonged exposure to the extract poses any significant health risks. Further research should explore a wider range of doses to establish a comprehensive dose–response relationship. This will aid in identifying the safe upper limits of the extract's administration and ensuring that even higher doses do not elicit toxic effects. Additionally, future studies should encompass a broader assessment of different organ systems, particularly those not covered in the initial study. A more comprehensive safety study requires expanding the range of toxicological endpoints to include immunotoxicity, genotoxicity, and reproductive toxicity. These endpoints will help identify any specific vulnerabilities across various physiological systems.

## 6. Conclusion

In this study, both acute and subacute toxicity tests of BAK cultivated in Morocco were evaluated. Through meticulous analysis, a total of 11 phenolic compounds were identified, with procyanidin and epicatechin emerging as the predominant constituents. The acute and subacute toxicity evaluations were carried out methodically, involving a series of rigorous tests and observations. The results were reassuring, showing no significant differences in key health indicators such as body weight, organ weight, and various hematological and biochemical parameters at the tested doses. Moreover, the detailed histological analysis of vital organs did not reveal any adverse effects, further supporting the safety profile of the studied extract. In order to fully understand the possible therapeutic uses of the identified phenolic compounds as well as to investigate the long-term consequences of ingestion, more research is necessary. Overall, this study contributes significantly to the existing body of knowledge on apricot kernels, offering a basis for further research and potential development of health-promoting products derived from these kernels. However, more extensive investigations should be conducted to comprehensively establish the safety profile of BAKs, by adopting additional toxicity assessment protocols, including chronic toxicity evaluations, studies with higher doses, and investigations across different species, besides the exploration of other extraction methods' effects on overall toxicity, notably due to the potential presence of cyanide.

## Figures and Tables

**Table 1:** Quantification of phenolic compounds of bitter apricot kernel extracts.

Order	Compounds	Aqueous extract	
		Tr (min)	Concentration in mg/g of the extract
1	Gallic acid	8.3	1.31 ± 0.02^a^
2	Vanillic acid	11.87	1.21 ± 0.82
3	Salicylic acid	13.27	0.33 ± 0.04
4	Syringic acid	13.9	2.72 ± 0.03
5	Resveratrol	15.7	2.15 ± 0.09
6	Chlorogenic acid	17.75	9.3 ± 0.11
7	Epicatechin	18.22	10 ± 0.54
8	Procyanidin	20.03	14.2 ± 0.96
9	Cyanidin 3-O-galactoside	21.89	8.7 ± 0.05
10	Mandelonitrile-beta-gentiobioside	33.9	4.1 ± 0.2
11	Tocopherol	34.6	6.29 ± 0.033
Total			60.31 ± 0.11

^a^Results correspond to mean ± standard deviation.

**Table 2:** Hematological parameters of mice treated with bitter apricot kernel extracts.

Hematological parameters	Control	100 mg/kg bw	500 mg/kg bw	1000 mg/kg bw
RBC (10^6^ μl^−1^)	10.24 ± 0.01	10.61 ± 0.78	10.73 ± 0.31	11.27 ± 1.64
HGB (g dL^−1^)	12.68 ± 0.74	12.92 ± 0.59	13.19 ± 0.54	11.48 ± 1.75
HCT (%)	42.67 ± 0.09	43.12 ± 0.06	42.98 ± 0.13	41.82 ± 1.01⁣^∗^
WBC (10^3^ μl^−1^)	13.07 ± 0.58	12.79 ± 0.30	13.91 ± 0.40	14.02 ± 0.12

*Note:* Results correspond to mean ± standard deviation and are considered statistically significant at ⁣^∗^*p* < 0.05.

**Table 3:** Biochemical parameters of mice treated with bitter apricot kernel extracts.

Biochemical parameters	Control	100 mg/kg bw	500 mg/kg bw	1000 mg/kg bw
AST (U/L)	139.5 ± 0.7	139 ± 1.41	141.5 ± 0.71	142.5 ± 0.71
ALT (U/L)	39.4 ± 0.7	39.5 ± 0.71	40.5 ± 3.54	40.5 ± 0.71
Urea (g/L)	0.28 ± 0.01	0.27 ± 0.06	0.3 ± 0.03	0.34 ± 0.02⁣^∗^
Creatinine (mg/L)	3.2 ± 0.42	3.2 ± 0.14	3.45 ± 0.07	3.7 ± 0.28⁣^∗^

*Note:* Results correspond to mean ± standard deviation and are statistically significant at ⁣^∗^*p* < 0.05.

## Data Availability

The data that support the findings of this study are available from the corresponding author upon reasonable request.
